# Cov-hep study: heparin in standard anticoagulation based on citrate for continuous veno-venous hemodialysis in patients with COVID-19: a structured summary of a study protocol for a randomized controlled trial

**DOI:** 10.1186/s13063-020-04814-0

**Published:** 2020-11-11

**Authors:** Paulo Ricardo Gessolo Lins, Claudia Coimbra César de Albuquerque, Camila Fernandes Assis, Bruna Cristine Duarte Rodrigues, Beatriz Pinto e Siqueira Campos, Eduardo de Oliveira Valle, Carla Paulina Sandoval Cabrera, Jeison de Oliveira Gois, Gabriela Cardoso Segura, Fernando Louzada Strufaldi, Lorena Catelan Mainardes, Rayra Gomes Ribeiro, Daniela Del Pilar Via Reque Cortes, Luciana Gil Lutf, Márcia Fernanda Arantes de Oliveira, Gabriel Teixeira Montezuma Sales, Igor Smolentzov, Bernardo Vergara Reichert, Lucia Andrade, Victor Faria Seabra, Camila Eleuterio Rodrigues

**Affiliations:** niversity of São Paulo – Hospital de Clínicas, São Paulo, Brazil

**Keywords:** COVID-19, Randomized controlled trial, protocol, Acute kidney injury, continuous dialysis, heparin, citrate, CVVHD, CVVHDF

## Abstract

**Objectives:**

The primary objective is to test if heparin added to a standard regional anticoagulation protocol based on citrate is able to reduce dialysis circuit losses by clotting without increasing the risk of thrombocytopenia or bleeding, in patients with COVID-19 with acute kidney injury requiring dialysis.

**Trial design:**

Randomized, parallel-group, open-label trial, with two arms (ratio 1:1) comparing different continuous renal replacement therapy anticoagulation strategies.

**Participants:**

Eligibility conditions:

All ICU patients of University of Sao Paulo General Hospital (Hospital das Clínicas), Brazil will be screened for eligibility conditions.

Adults (> 18 years old) with confirmed COVID-19 and acute kidney injury requiring dialysis with agreement between ICU and nephrology teams for the introduction of renal continuous replacement therapy in daily ICU rounds. Continuous renal replacement therapy will be prescribed by consulting nephrologists based on standard clinical guidelines, including acute kidney injury with hemodynamic instability plus hyperkalemia, severe acidosis, volume overload, respiratory distress, multiorgan failure or some combination of these factors.

Data Collection:

Patients demographics and associated clinical data and comorbidities will be recorded at ICU entry. Demographic information will include the patient’s age, sex, and admission dates. Clinical data comprise comorbidities, APACHE 2, SAPS 3, need for mechanical ventilation, and use of vasopressor drugs. Physiological data collected by the day of CRRT start will be vital signs, the arterial oxygen tension/fraction of inspired oxygen (PaO2/FiO2) index, and serum creatinine, blood urea nitrogen, bilirubin, hemoglobin, hematocrit, platelets, white blood cell count levels and Peak D-dimer levels.

Patients will be analyzed for the first 72h of CRRT, and they will be evaluated regarding clinical variables, filter patency and any adverse events that could be related to the anticoagulation choice, as bleeding (mild or major) or low platelets counts (<100.000 ui/uL) during treatment period. Mild and major bleeding will be defined by hemorrhagic event without clinical impact or hemoglobin (Hb) fall lesser than 1g/dL and hemorrhagic event with clinical impact or Hb fall higher than 1g/dL, respectively.

Exclusion criteria:

Hypersensitivity to any of the substances going to be used in the study (Citric acid dextrosol 2.2% and unfractionated heparin); Previous diagnosis of coagulopathy or thrombophilia; Contraindication to the use of unfractionated heparin; Risk of citrate poisoning - (Lactate> 30 mg/dL, international normalized ratio > 2.5, Total bilirubin> 15 mg/dL); Pregnancy; Patients unlikely to survive for more than 24 hours.

The trial is being undertaken at the University of Sao Paulo General Hospital (Hospital das Clinicas), Brazil.

**Intervention and comparator:**

**Group A (control) -** Patients on continuous renal replacement therapy (blood flow 150 ml/min, dose of 30 mL/Kg/h) receiving anticoagulation with sodium citrate at 4 mmol/L

**Group B (experiment):** Patients on continuous hemodialysis (blood flow 150 mL/min, dose of 30 mL/Kg/h) receiving anticoagulation with sodium citrate at 4 mmol/L associated with unfractionated heparin at 10 U/Kg/h.

**Main outcomes:**

The percentage of clotted dialyzers within 72 hours in each of the studied groups (Primary outcome)

Secondary outcomes: Number of dialyzers used in the first 72 hours of dialysis protocol, Mortality in the first 72 h of dialysis protocol, Bleeding events (Major or minor) in the first 72 h of dialysis protocol, Thrombocytopenia (less than 50.000 platelets) proportion in the first 72 h of dialysis protocol, Dialysis efficiency (Urea sieving) - variation in urea sieving between the first, second and third days of dialysis protocol, Continuous renal replacement therapy pressures (Arterial, Venous, dialysate and pre-filter pressure) in the first 72 h of dialysis protocol, in-hospital mortality.

**Randomization:**

RedCap→ randomization – 2 blocks randomization by D-dimer level (5000ng/dL cut-off) and catheter site (Right Internal Jugular versus other sites) with 1:1 allocation ratio.

**Blinding (masking):**

No blinding – Open label format

**Numbers to be randomized (sample size):**

Total number of patients 90 (45 per group)

**Trial Status:**

Trial version 2.0 – ongoing recruitment.

First recruitment: June 29, 2020

Estimated date for last recruitment: December 31, 2020

**Trial registration:**

Responsible Party: University of Sao Paulo General Hospital (Hospital das Clinicas)

ClinicalTrials.gov Identifier: NCT04487990, registered July 27, 2020, ReBec www.ensaiosclinicos.gov.br/rg/RBR-45kf9p/

Other Study ID Numbers: U1111-1252-0194

**Full protocol:**

The full protocol is attached as an additional file, accessible from the Trials website (Additional file 1) In the interest in expediting dissemination of this material, the familiar formatting has been eliminated; this Letter serves as a summary of the key elements of the full protocol.

## Supplementary information

**Additional file 1.** Full protocol

## Data Availability

All the Steering Committee members and co-authors will have access to the original dataset. The data will be available from the author on reasonable request by email.

