# Supplementary material for: Cov-hep study: heparin in standard anticoagulation based on citrate for continuous veno-venous hemodialysis in patients with COVID-19: a structured summary of a study protocol for a randomized controlled trial
Source: Trials. 2020 Nov 11;21:920. doi: 10.1186/s13063-020-04814-0 (PMC7656196; doi:10.1186/s13063-020-04814-0)
Supplement: Supplementary file 1 — Additional file 1. Full protocol [file 13063_2020_4814_MOESM1_ESM.pdf]

MEDICINA
USP

ESTUDO CoV-Hep: Ensaio clínico randomizado, duplo cego, pareado comparando modalidades de anti-coagulação regional em hemodiálise veno-venosa continua em portadores de COVID-19

Disciplina de Nefrologia, Departamento de Clínica Médica, FMUSP

Maio, 2020

MEDICINA
USP

Av. Dr. Arnaldo, 455 · Cerqueira César
São Paulo · Brasil · 01246 903

MEDICINA
USP

Resumo

Desde o surgimento da nova cepa de betacoronavírus (SARS-CoV-2) e suas repercussões clínicas exasperadas, tem sido descrito que pacientes portadores da respectiva pneumonia (COVID-19) apresentam taxas elevadas de eventos trombóticos, inclusive redução do tempo de meia vida dos capilares quando submetidos a terapia renal substitutiva. São descritas diversas estratégias de prevenção da perda precoce de capilares de diálise, sendo a anticoagulação regional baseada em infusão de soluções de citrato a modalidade preferencial de prevenção dessa complicação. Por outro lado, em pacientes portadores de SARS-CoV-2 já existem descrições da presença de inflamação endotelial e ativação da cascata da coagulação de maneira desenfreada, inclusive com estudos que demonstram o benefício da heparinização destes pacientes. Sendo assim, esse estudo visa a comparar duas diferentes estratégias de anticoagulação em pacientes infectados por COVID-19 com necessidade hemodiálise veno-venosa continua. A partir da indicação da terapia renal substitutiva contínua, os pacientes serão triados pelos critérios de elegibilidade e, caso se enquadrem em tais parâmetros, serão randomizados em dois grupos: Grupo A – Anticoagulação regional padrão baseada em Citrato associado a infusão de doses baixas de heparina não fracionada na titulação de 10ui/kg/hora e Grupo B – Anticoagulação regional padrão baseada em Citrato e infusão de solução placebo. Os pacientes serão randomizados em blocos e acompanhados por 72 horas. O objetivo primário é a avaliação da patência do capilar ao final de 72 horas de seguimento clínico. Objetivos secundários serão mortalidade, taxa de sangramento, queda dos índices hematimétricos, *sieving* de ureia, tempo do filtro em horas, tempo com terapia desligada (*down time*), valores de pressões do capilar dialisador (PBE e PTM). Todos os pacientes serão submetidos a um procedimento padrão com dose prescrita de 30mL/Kg/H, fluxo de sangue de 150mL/minuto e mesmo capilar (polisulfona).

MEDICINA

USP

Situação do COVID-19 no mundo

Em meados de dezembro de 2019, inúmeros casos de uma pneumonia de origem até então desconhecida foram descritos na cidade de Wuhan, província de Hubei, na China. Estudos de sequenciamento demonstraram que se tratava de um novo betacoronavírus, que posteriormente foi denominado como SARS-CoV-2 (1-3). Após identificação do vírus em outros países como Japão, Singapura, Tailândia e República de Coréia, em 30 de janeiro, a OMS declarou o COVID-19 como emergência de saúde pública internacional, reconhecendo a presença do mesmo em quase 5 regiões no período de 1 mês, acreditando que a detecção precoce e o distanciamento social, ajudariam a conter a transmissão do vírus. Em 7 de março, 100.000 casos foram reportados em 100 países do mundo, sendo considerado o vírus como uma ameaça pela rápida propagação, sendo que no dia 11 de março, a OMS declarou o COVID - 19 como pandemia. Em 13 de março, a Europa se tornou o epicentro da pandemia, tendo mais casos reportados e mortes em comparação com o resto do mundo (4). Em 16 de março, a OMS indicou a realização de testes em pessoas sintomáticas e em aquelas que estiveram em contato com casos positivos, e a instauração de medidas de distanciamento social.

No Brasil, o enfrentamento do coronavírus começou em fevereiro, com a repatriação de 34 brasileiros que moravam em Wuhan, sendo orientados a permanecer em quarentena por 14 dias na Base Aérea de Anápolis, no estado de Goiás. Em 26 de fevereiro, se confirmou o primeiro caso de COVID-19 em São Paulo. Em 17 de março é notificada a primeira morte por coronavírus, e em 20 de março se declara a transmissão como comunitária. Até o presente momento (04/05/2020), foram notificados 102 mil casos confirmados e 7051 óbitos.

MEDICINA USP

Quadro clínico e lesão renal aguda

Em um estudo recente contemplando 5700 pacientes hospitalizados devido a infecção pelo COVID-19, 30% dos pacientes apresentavam-se febris, enquanto taquipneia (frequência respiratória maior que 24 inspirações por minuto) estava presente em 17% e 28% necessitaram de suplementação de oxigênio. Dessa coorte de pacientes, 14% necessitaram de internação em unidade de terapia intensiva e 22% apresentaram critérios de lesão renal aguda (LRA). Dentro do grupo que necessitou de terapia intensiva, 86% necessitaram de ventilação invasiva, e 25% de terapia renal substitutiva (5). Nesta coorte, lesão renal aguda foi definida de acordo com a classificação de KDIGO para LRA (Figura 1) (6).

	CREATININA SÉRICA	DIURESE
ESTÁGIO 1	1,5 a 1,9 vezes o basal em 1 SEMANA<br>Ou<br>Aumento ≥ 0,3mg/dL em 48 HORAS	< 0,5 mL/Kg/H por 6-12 horas
ESTÁGIO 2	2,0 a 2,9 vezes o basal em 1 SEMANA	< 0,5mL/Kg/H por ≥ 12 horas
ESTÁGIO 3	3,0 vezes o basal em 1 SEMANA<br>ou<br>Aumento acima de 0,3mg/dL em Creatinina ≥ 4,0mg/dL<br>ou<br>Inicio de Terapia dialítica	< 0,3mL/Kg/H por ≥ 24 horas<br>Ou<br>Anúria por ≥ 12 horas

Figura 1 - Classificação da Lesão Renal Aguda de acordo com os critérios de KDIGO

Até o momento, não existe um tratamento específico contra o novo coronavírus, porém, vários medicamentos estão sendo investigados em ensaios clínicos, e até obter um resultado deles, a atenção para os pacientes com COVID - 19 é principalmente de suporte, especialmente em ambiente de UTI. Nos pacientes com disfunção renal, a terapia de substituição renal (TSR) é mais uma ferramenta que faz parte do manejo do

MEDICINA USP

paciente. Segundo a experiência em Seattle com pacientes com IRA e COVID-19, a necessidade de diálise aparece na segunda semana após iniciados os sintomas, e representa 5% dos pacientes da UTI. A experiência do grupo de LRA do HCFMUSP mostra um número bem maior – até 50% dos pacientes internados em UTI desde o início do recebimento dos casos de pacientes com COVID-19 em nosso serviço têm necessidade de TSR. No referente à escolha do melhor método dialítico, ainda não se dispõe de evidência científica suficiente de qual método é superior; a sugestão é escolher o tipo de diálise de acordo com a gravidade da doença, levando em conta a disponibilidade dos recursos e a experiência de cada centro de saúde (7).

COVID-19 e hipercoagulabilidade

Em quadros mais graves de COVID-19 o dano endotelial com tromboses subsequentes tem sido reportado, inclusive com elevações de níveis séricos de dímero-D como marcador de pior prognóstico (8). Relatos de coagulopatia associada a níveis elevados de anticorpos antifosfolípides (9) têm surgido, assim como de acidentes vasculares cerebrais isquêmicos em populações jovens (10), infarto agudo do miocárdio (11) e tromboembolismos pulmonares (12). Alguns estudos de análise retrospectiva sugerem que o uso de heparina possa ser benéfico em pacientes com COVID-19 grave (13), mas este dado ainda precisa de confirmação científica mais rigorosa. Alguns centros têm preconizado anticoagulação plena para os pacientes com COVID-19, a despeito da baixa evidência de benefício até o momento.

Anticoagulação regional em terapia renal substitutiva

Um grupo italiano de nefrologistas, "Brescia Real Covid Task Force", produziu um documento de diretrizes que sugere algumas condutas baseadas na experiência inicial de alguns serviços em relação ao manejo da COVID-19 (14). Neste documento, endossado pela Associação Renal Européia – Associação Européia de Diálise e

MEDICINA USP

Transplante (ERA-EDTA), sugere-se que a anticoagulação das terapias de substituição renal contínuas seja feita primariamente com citrato regional, seguindo a recomendação das diretrizes do KDIGO para LRA em situações gerais (6). A segunda opção de anticoagulação seria a anticoagulação sistêmica com heparina não-fracionada para um TTPa entre 1,0 a 1,4 vezes o valor da normalidade. Este documento serviu como base para as recomendações de anticoagulação para terapias contínuas da Sociedade Internacional de Nefrologia (ISN).

Existem alguns relatos que sugerem que a situação de hipercoagulabilidade leve a maior perda dos filtros e circuitos extracorpóreos, com necessidade mais frequente de troca de sistemas numa época de grande preocupação com insumos hospitalares (15). A Sociedade Brasileira de Nefrologia (SBN), em posicionamento conjunto com a Associação de Medicina Intensiva Brasileira sugere que em terapias contínuas cada serviço use seu protoloco usual de anticoagulação, com recomendação de que não haja terapias sem o uso de anticoagulantes. Sugere-se primariamente a adequação do acesso vascular, com possibilidade de intensificar a anticoagulação com aumento de dose de heparina ou, quando da anticoagulação com citrato, com redução do objetivo de cálcio iônico pós-capilar (16).

A análise subjetiva dos pacientes internados no HCFMUSP desde o início das admissões dos pacientes com COVID-19 também sugere coagulação mais precoce e frequente dos capilares dialisadores e linhas, o que fez com que a conduta frente a esses pacientes fosse diferente dos pacientes previamente avaliados: o protocolo prévio de anticoagulação nos pacientes em hemodiálise veno-venosa continua (CVVHD) constava de uso de citrato regional em dose de 3 mmol/litro de sangue tratado, com objetivo de cálcio iônico pós-capilar entre 1,0 e 1,4 mg/dl. Após essa análise subjetiva de maior índice de perdas de sistemas, o protocolo assistencial que passou a ser adotado foi o uso de citrato regional em dose de 4 mmol/litro de sangue tratado, com objetivo de cálcio iônico pós-capilar menor que 1,0 mg/dl associado a heparina não-fracionada em dose de 10ui/Kg/H. Entretanto, a custo-efetividade desta alteração não foi avaliada, e este é o principal objetivo do estudo que aqui se apresenta.

Objetivos

MEDICINA USP

Desfecho primário: Comparar taxa de patência do filtro em 72 horas em terapia renal substitutiva continua utilizando uma das estratégias de anticoagulação do estudo.

Desfechos secundários:

Comparar tempo de patência do filtro até o tempo final de 72 horas
Número de filtros utilizados nas 72 horas
Avaliar as taxas de sangramento e quedas hematimétricas
Avaliar queda no número de plaquetas
Avaliar as diferenças de pressões no filtro durante as 72 horas de tratamento
Avaliar o *sieving* da ureia durante as 72 horas de terapia
Avaliar tempo com terapia desligada
Comparar taxas de coagulação de filtro entre as diferentes modalidades de terapia empregadas
Avaliar a mortalidade

MEDICINA

USP

Planejamento do estudo

Fase observacional:

Além do estudo principal, de intervenção, objetiva-se realizar uma análise observacional retrospectiva do que já foi feito em relação à anticoagulação em terapias contínuas recentemente em nosso serviço.

Serão avaliados todos os pacientes submetidos a CVVHD com o uso de citrato a 3 mmol/litro de sangue tratado durante o mês de setembro de 2019 (6 meses anteriores ao início dos casos de COVID-19 no Brasil), com análise de cada dia sob terapia de cada paciente individualmente. Serão analisados o número de terapias desligadas por coagulação do sistema, a quantidade de trocas de insumos, as pressões de acesso e de capilar (PA, PV, PBE e PTM), o calibre do catéter utilizado, o tempo de cálcio iônico pós-capilar acima do alvo e os coagulogramas dos pacientes.

Esses pacientes serão comparados em relação aos parâmetros estudados aos pacientes com diagnóstico de COVID-19 e submetidos a CVVHD com o uso de citrato a 3 a 4 mmol de citrato/litro de sangue tratado (pacientes inicialmente avaliados com COVID-19, antes da alteração do protocolo assistencial, e pacientes com contra-indicação a heparina).

O terceiro grupo analisado será o de pacientes com COVID-19 submetidos a CVVHD com uso de citrato em dose de 4 mmol/litro de sangue tratado associado a heparina não-fracionada em dose de 10ui/Kg/H.

Fase de intervenção:

Delineamento

Estudo clínico, randomizado, com alocação em blocos, de superioridade, com avaliação cega dos desfechos e análise por intenção de tratar.

Elegibilidade

Critérios de Inclusão

Homens e mulheres com idade superior a 18 anos;

Infecção confirmada ou provável por SARS-CoV-2

Presença de lesão renal aguda com indicação e concordância entre equipe assistente e de suporte nefrológico de introdução de terapia renal substitutiva – modalidade contínua – tipo hemodiálise veno-venosa continua.

Critérios de Exclusão

Hipersensibilidade a qualquer uma das substâncias utilizadas no estudo (Ácido cítrico dextrosol 2,2% e Heparina não fracionada)

Diagnóstico prévio de coagulopatia ou trombofílias

Contra-indicação ao uso de heparina não fracionada pela equipe assistente

Risco de intoxicação por citrato – (Lactato > 30mg/dL, IRN > 2.5, Bilirrubina total > 15mg/dL)

Gestantes

Critérios diagnósticos de infecção por COVID-19

Os critérios para diagnóstico serão baseados no Guia da Organização Mundial de Saúde, onde os casos confirmados de infecção por SARS-CoV-2 serão aqueles realizados por testes moleculares com PCR quantitativo em tempo real (RT-PCR) de amostras de swabs nasofaringial, lavados broncoalveolar, aspirado traqueal nasofaringe ou teste orientado pela OMS com validade diagnóstica (17, 18).

Os casos prováveis serão aqueles, em que os indivíduos que apresentem critérios orientados pelo Ministério da Saúde e Organização Mundial da Saúde, como: deslocamentos a regiões com alto índice de indivíduos infectados; contato prévio com outras pessoas com confirmações de infecção por SARS-CoV2 em menos de 14 dias, e

que apresentem sintomas de vias aéreas superiores e/ou inferiores (coriza, irritação ocular, tosse seca ou produtiva, falta de ar, etc) associado a imagem radiográfica compatível (infiltrado intersticial na radiografia de tórax e/ou infiltrado em vidro fosco em tomografia) (19).

Intervenções do Estudo

A prescrição da terapia renal substitutiva será realizada conforme padrão institucional, com o aparelho de diálise continua disponível para uso na instituição (Diapact BBRAUN, Omni BBRAUN, PrismaFlex Baxter ou Multifiltrate Fresenius)– modalidade CVVHD (Hemodiálise veno-venosa contínua) na dose de 30mL/Kg/H (Dose máxima de 3000mL/H) ou CVVHDF (Hemodiafiltração veno-venosa contínua) na dose de 30ml/kg/H fracionadas em 1/3 de dose de filtração e 2/3 de dose de diálise, filtro de alta permeabilidade compatível com o maquinário e fluxo de sangue 150mL/min.

A anticoagulação regional será avaliada no presente estudo, utilizado para tal fim uma solução padrão de ácido cítrico dextrosol 2,2% na dose de 4mmol de citrato para cada litro de sangue tratado – infusão calculada de 300mL/H associado a terapia estudada – Solução A – Solução de heparina não fracionada na diluição de 100ui/kg com dose prescrita de 10ui/Kg/H ou Solução B – Solução de soro fisiológico (Placebo) na dose de 0,1mL/kg/H.

Tabela de correção para Anticoagulação regional com HNF:

TTPa	Meta
1.0 – 2.0	Manter infusão
2.0 – 3.0	Pausar bomba de infusão por 1 hora. Reduzir dose da Bomba de Infusão em 2mL/H
> 3.0	Pausar bomba de infusão por 2 horas, Recoletar TTPa: Se TTPa < 2,0 – Reiniciar infusão reduzindo dose em 4mL/H. Se TTPa > 2,0 – Manter Bomba de infusão desligada por mais 2 horas.

Randomização

Os pacientes que atenderem a todos os critérios de elegibilidade serão randomizados em uma proporção 1:1 para o grupo intervenção (Solução A ou B). A sequência de randomização será gerada por um software validado com blocos de tamanho variável. Esta randomização será realizada no sistema RedCap.

Plano de coleta de dados

Parâmetros	Hora 0	Hora 12	Hora 24	Hora 36	Hora 48	Hora 60	Hora 72
**Avaliação clínica inicial**	■						
**Laboratório Inicial**	■						
**Laboratório de segurança da terapia**		■	■	■	■	■	■
**Pressões da terapia**		■	■	■	■	■	■
**Sieving de ureia**			■		■		■

Parâmetros de coleta

Caso o paciente seja alocado no estudo, os parâmetros abaixo serão coletados de forma prospectiva em plataforma padronizada (RedCAP).

Avaliação Clínica Inicial:

Sexo, Idade, Peso na admissão, Altura, Data da Internação Hospitalar, Data do Início dos Sintomas, Data da Internação em UTI, Ventilação Mecânica, Droga Vasoativa, Inotrópicos, Posição Prona, Etiologia da LRA, Creatinina referência, APACHE 2, SAPS 3

MEDICINA USP

Laboratório Inicial:

Hemograma completo, TTPa, RNI, Dímero-D, DHL, CPK, Albumina, Creatinina, Ureia, Sódio, Potássio, Gasometria, Lactato, Fósforo, Cálcio total, Cálcio Iônico, Urina tipo 1, Bilirrubina total e frações, TGO, TGP

Laboratório de Segurança:

Hemograma, TTPa, Cálcio iônico pós Filtro, Cálcio iônico sistêmico, Cálcio Total Sistêmico, Ureia, Potássio, Gasometria, Lactato, RNI

Pressões da terapia:

Pressão Arterial, PBE, PTM, Pressão Venosa, Pressão Dialisato

Dispensa de Termo de Consentimento Livre Esclarecido (TCLE)

Considerando que tanto a anticoagulação com heparina como a anticoagulação regional com citrato são recomendadas na literatura médica como formas de anticoagulação na terapia renal substitutiva contínua (14), e que ambas já são utilizadas, inclusive em associação, como protocolos assistenciais na rotina diária de alguns serviços, incluindo o nosso serviço de nefrologia do HCFMUSP, e sabendo da baixíssima taxa de complicações relacionada a anticoagulação regional com heparina não fracionada e/ou citrato (20) além da premissa da dificuldade logística imposta as restrições de acesso de familiares no Instituto Central – Hospital de Clínicas – FMUSP e não possibilidade de aplicar o TCLE nos pacientes devido a estarem inconscientes, solicitamos a isenção da aplicação do TCLE.

Reforçamos que o investigador manterá um registro de identificação dos participantes da pesquisa e que seus registros poderão ser acessados por autoridades sanitárias e pela equipe autorizada do centro coordenador sem violação da confidencialidade do participante da pesquisa, no nível permitido pela lei ou regulamentação aplicável.

MEDICINA USP

Análise estatística

A análise primária será baseada em uma população com intenção de tratar, incluindo participantes randomizados. As análises de segurança serão baseadas em uma população modificada de intenção e tratamento, composta por todos os participantes que receberam pelo menos 24 horas da terapia proposta.

Este é um estudo randomizado, controlado, prospectivo, com controle ativo que testa uma hipótese de superioridade com uma taxa de erro do tipo I bilateral de 0,05. Hipóteses secundárias foram ordenadas de acordo com a importância relativa. As características clínicas da população serão descritas de acordo com as estatísticas de resumo apropriadas (por exemplo, proporções para dados categóricos, médias com intervalos de confiança de 95% para dados contínuos, mediana para dados de tempo até o evento).

Cálculo amostral

Consideramos dados do próprio grupo e literatura pertinente (21-23) – existem descrições de que a taxa de sobrevida média em horas de filtros utilizando citrato como anticoagulação regional situa-se na faixa de 50 horas com taxas de coagulação em 72 horas próximas a 60%. Considerando um poder estatístico de 80% e um erro alfa de 0,05 e uma taxa de perda de 15%, serão necessários 45 pacientes em cada grupo para detecção de uma diferença de 50% na taxa de filtros coagulados em 72 horas.

Riscos

A condição de saúde do participante pode permanecer a mesma, melhorar ou piorar, e existe a possibilidade de ocorrerem efeitos adversos durante o estudo. Os procedimentos que serão realizados durante todo o período da pesquisa poderão apresentar alguns riscos mínimos relacionados às coletas laboratoriais (já rotineiras nas unidades), incluindo, mas não limitado a possíveis pequenos hematomas devido à coleta de amostras de sangue. No uso da heparina não fracionada, os efeitos mais comuns são: sangramentos e trombocitopenia, principalmente. No caso do citrato os participantes poderão apresentar alguns efeitos colaterais do seu acúmulo tais como: hipocalcemia e

desarranjos ácido-básico. Para evitar tais riscos, haverá um acompanhamento intensivo do paciente na unidade hospitalar que serão realizadas no segmento do estudo. Além disso, as doses utilizadas no estudo se mostraram relativamente seguras e amplamente utilizadas na prática clínica de anticoagulação regional de circuitos de terapias extracorpóreas (22, 24).

Avaliações feitas por profissionais treinados e quaisquer alterações serão identificadas e tomadas as devidas ações de minimização e/ou recuperação. Além disso, o participante terá acesso e orientações quanto a todos os exames realizados. A qualquer momento, o participante poderá ter acesso a todas as informações obtidas a seu respeito neste estudo ou a respeito dos resultados gerais do estudo. A desistência e/ou término do estudo não afetará a assistência médica oferecida ao participante.

Qualquer intercorrência de saúde derivada do estudo será avaliada e acompanhada por profissionais da equipe de saúde. Se uma lesão ou qualquer dano à saúde ocorrer como comprovado resultando da participação na pesquisa, assistência integral estará disponível pelo período que for necessário, sem que o participante tenha gastos.

As pesquisas são avaliadas e aprovadas pelo Comitê de Ética em Pesquisa (CEP) para garantir que os direitos e bem-estar dos participantes da pesquisa sejam protegidos. Toda a informação médica será sigilosa e somente a equipe do estudo terá acesso para evitar quebra de confidencialidade. Em nenhum momento, o nome do participante ou qualquer informação sobre a sua saúde será fornecida para qualquer pessoa que não seja da equipe do estudo. As informações serão confidenciais e utilizadas somente para fins desta pesquisa. Todas as medidas cabíveis para evitar a quebra de sigilo da identidade do participante e confidencialidade serão executadas. Estas incluem acesso a documentos do estudo somente às pessoas da equipe de pesquisa, armazenamento de documentos impressos em arquivo trancado em local com acesso restrito, e armazenamento de dados eletrônicos em uma base de dados com acesso seguro por meio de usuário e senha individuais somente para os pesquisadores e outros membros da equipe de pesquisa. Os resultados do estudo serão divulgados, para fins acadêmicos e científicos, sem a identificação de nenhum dado que revele a identidade dos participantes.

Benefícios

MEDICINA
USP

Não há nenhuma terapia aprovada ou evidência robusta sobre qualquer terapia para o tratamento da infecção por SARS-CoV2 ou suas complicações diretas e indiretas. Até o momento parte das ações terapêuticas são tomadas por ideias empíricas, ou apenas com base em achados laboratoriais, pois o momento de pandemia requer ações rápidas e estudos com o objetivo de buscar evidências de eficácia que possam ser rapidamente aplicadas aos demais indivíduos acometidos, neste caso da infecção. Poderá não haver benefícios na participação no estudo, mas de qualquer sorte julgamos fundamental e prioritária a busca por uma opção terapêutica eficaz e segura de forma que a condução de um estudo randomizado nos moldes da presente proposta se faz necessário.

CRONOGRAMA DE ATIVIDADES

	Mai/20	Jun/20	Jul/20	Ago/20	Set/20	Out/20	Nov/20	Dez/20	Jan/21
**Inclusão de pacientes**									
**Análise estatística**									
**Redação de papers**									

Bibliografia:

1. Chan JF, Yuan S, Kok KH, To KK, Chu H, Yang J, et al. A familial cluster of pneumonia associated with the 2019 novel coronavirus indicating person-to-person transmission: a study of a family cluster. Lancet. 2020;395(10223):514-23.
2. Zhu N, Zhang D, Wang W, Li X, Yang B, Song J, et al. A Novel Coronavirus from Patients with Pneumonia in China, 2019. New England Journal of Medicine. 2020;382(8):727-33.
3. Huang C, Wang Y, Li X, Ren L, Zhao J, Hu Y, et al. Clinical features of patients infected with 2019 novel coronavirus in Wuhan, China. Lancet. 2020;395(10223):497-506.
4. Mavragani A. Tracking COVID-19 in Europe: Infodemiology Approach. JMIR Public Health Surveill. 2020;6(2):e18941.
5. Richardson S, Hirsch JS, Narasimhan M, Crawford JM, McGinn T, Davidson KW, et al. Presenting Characteristics, Comorbidities, and Outcomes Among 5700 Patients Hospitalized With COVID-19 in the New York City Area. JAMA. 2020.
6. Khwaja A. KDIGO clinical practice guidelines for acute kidney injury. Nephron Clin Pract. 2012;120(4):c179-84.
7. Durvasula R, Wellington T, McNamara E, Watnick S. COVID-19 and Kidney Failure in the Acute Care Setting: Our Experience From Seattle. Am J Kidney Dis. 2020.
8. Tang N, Li D, Wang X, Sun Z. Abnormal coagulation parameters are associated with poor prognosis in patients with novel coronavirus pneumonia. J Thromb Haemost. 2020;18(4):844-7.
9. Zhang Y, Xiao M, Zhang S, Xia P, Cao W, Jiang W, et al. Coagulopathy and Antiphospholipid Antibodies in Patients with Covid-19. New England Journal of Medicine. 2020;382(17):e38.
10. Oxley TJ, Mocco J, Majidi S, Kellner CP, Shoirah H, Singh IP, et al. Large-Vessel Stroke as a Presenting Feature of Covid-19 in the Young. New England Journal of Medicine. 2020:e60.
11. Bangalore S, Sharma A, Slotwiner A, Yatskar L, Harari R, Shah B, et al. ST-Segment Elevation in Patients with Covid-19 — A Case Series. New England Journal of Medicine. 2020.
12. Poissy J, Goutay J, Caplan M, Parmentier E, Duburcq T, Lassalle F, et al. Pulmonary Embolism in COVID-19 Patients: Awareness of an Increased Prevalence. Circulation.0(0).
13. Tang N, Bai H, Chen X, Gong J, Li D, Sun Z. Anticoagulant treatment is associated with decreased mortality in severe coronavirus disease 2019 patients with coagulopathy. J Thromb Haemost. 2020.
14. Alberici F, Delbarba E, Manenti C, Econimo L, Valerio F, Pola A, et al. Management Of Patients On Dialysis And With Kidney Transplant During SARS-COV-2 (COVID-19) Pandemic In Brescia, Italy. Kidney Int Rep. 2020:10.1016/j.ekir.2020.04.001.

15. Burgner A, Ikizler TA, Dwyer JP. COVID-19 and the Inpatient Dialysis Unit. Managing Resources During Contingency Planning Pre-Crisis. 2020:CJN.03750320.
16. Nefrologia SBd. Nota técnica e orientações sobre a injúria renal aguda (IRA) em pacientes com COVID-19. 2020.
17. Zhai P, Ding Y, Wu X, Long J, Zhong Y, Li Y. The epidemiology, diagnosis and treatment of COVID-19. Int J Antimicrob Agents. 2020:105955-.
18. Ahn DG, Shin HJ, Kim MH, Lee S, Kim HS, Myoung J, et al. Current Status of Epidemiology, Diagnosis, Therapeutics, and Vaccines for Novel Coronavirus Disease 2019 (COVID-19). J Microbiol Biotechnol. 2020;30(3):313-24.
19. Brasil MdSd. PROTOCOLO DE MANEJO CLÍNICO DO CORONAVÍRUS (COVID-19) NA ATENÇÃO PRIMÁRIA À SAÚDE.
20. Claure-Del Granado R, Macedo E, Soroko S, Kim Y, Chertow GM, Himmelfarb J, et al. Anticoagulation, delivered dose and outcomes in CRRT: The program to improve care in acute renal disease (PICARD). Hemodial Int. 2014;18(3):641-9.
21. Schilder L, Nurmohamed SA, Bosch FH, Purmer IM, den Boer SS, Kleppe CG, et al. Citrate anticoagulation versus systemic heparinisation in continuous venovenous hemofiltration in critically ill patients with acute kidney injury: a multi-center randomized clinical trial. Crit Care. 2014;18(4):472.
22. Gattas DJ, Rajbhandari D, Bradford C, Buhr H, Lo S, Bellomo R. A Randomized Controlled Trial of Regional Citrate Versus Regional Heparin Anticoagulation for Continuous Renal Replacement Therapy in Critically Ill Adults. Crit Care Med. 2015;43(8):1622-9.
23. Kalb R, Kram R, Morgera S, Slowinski T, Kindgen-Milles D. Regional citrate anticoagulation for high volume continuous venovenous hemodialysis in surgical patients with high bleeding risk. Ther Apher Dial. 2013;17(2):202-12.
24. Raymakers-Janssen PAMA, Lilien M, van Kessel IA, Veldhoen ES, Wösten-van Asperen RM, van Gestel JPJ. Citrate versus heparin anticoagulation in continuous renal replacement therapy in small children. Pediatr Nephrol. 2017;32(10):1971-8.
